# Comment on Svarch-Pérez et al. Methods for a Non-Targeted Qualitative Analysis and Quantification of Benzene, Toluene, and Xylenes by Gas Chromatography-Mass Spectrometry of E-Liquids and Aerosols in Commercially Available Electronic Cigarettes in Mexico. *Int. J. Environ. Res. Public Health* 2024, *21*, 1308

**DOI:** 10.3390/ijerph22071049

**Published:** 2025-06-30

**Authors:** Roberto A. Sussman, Humberto Gómez-Ruiz, Konstantinos Farsalinos

**Affiliations:** 1 Institute of Nuclear Sciences, National Autonomous University of Mexico (UNAM), Alcaldía de Coyoacán, Mexico City 04510, Mexico; 2 Department of Analytical Chemistry, Faculty of Chemistry, National Autonomous University of Mexico (UNAM), Alcaldía de Coyoacán, Mexico City 04510, Mexico; hgomezruiz@gmail.com; 3 Laboratory of Molecular Biology and Immunology, Department of Pharmacy, University of Patras, 26500 Rio-Patras, Greece; kfarsalinos@gmail.com

The authors of a study recently published in IJERPH quantified levels of benzene, toluene and xylenes (BTXs) in e-liquids and aerosols in a sample of 20 disposable e-cigarettes collected in Mexico City [1]. They conclude that these concentrations exceed the concentrations of these compounds given by the Threshold Limit Values (TLVs), Permissible Exposure Limit (PEL) and Short-Term Exposure Limit (STEL) of the California Occupational Safety and Health Administration (Cal/OSHA) [2].

This article exhibits severe methodological flaws and serious analytical and interpretive errors that invalidate its findings and render its conclusions misleading.

The major issues, addressed in detail below, are the following:

1. The authors erroneously converted Cal/OSHA PEL values from ppm to µg/L by multiplying the ppm by 1000, overlooking the established conversion formula that considers the molecular weight of a substance in order to make the conversion. They also failed to notice that the Cal/OSHA PEL document, which they cite, had already included values in mg/m^3^ (which is the same value when expressed in μg/L) for toluene and xylenes [2].
2. They compared e-cigarette BTX emissions in µg/L of aerosolized e-liquid with Cal/OSHA PELs expressed in µg/L of ambient air, which is scientifically meaningless. We note that 1 L of ambient air corresponds to two breaths, while 1 L of aerosolized liquid corresponds to 200–250 days of e-cigarette use, as explained below.
3. The presentation of the comparison with tobacco cigarettes is also wrong because their source reported the values in ppb [3]. Therefore, the same error as with PELs was made in converting the values to μg/L. Furthermore, there is no scientific basis in comparing values in tobacco cigarettes that represent the amount per L of smoke inhaled (representing fewer than two cigarettes, as explained below) with values in e-cigarettes that represent the amount per L of liquid aerosolized (representing 200–250 days of consumption).
4. Even beyond occupational settings, BTXs are ubiquitous in an indoor environment, and the recommended exposure limits for indoor spaces (just breathing air at home) result in far higher exposure from breathing air at home than from e-cigarette use, as calculated below.

In detail, it is well-established that concentrations expressed in ppm in ambient air can be converted to mass per volume of air only after considering the molecular weight of each compound, using the formula [4]

μg/L = (ppm × molecular weight)/24.45

In fact, the Cal/OSHA document, which Svarch-Pérez et al. cite in their article [2], already provides both ppm and mg/m^3^ (which the latter being the same value when expressed in μg/L) for toluene (10 ppm, 37 mg/m^3^) and xylenes (100 ppm, 435 mg/m^3^). The mg/m^3^ (i.e., μg/L) values reported in the document are in agreement with the formula provided above and, obviously, are very different from the conversion made by Svarch-Pérez et al. Therefore, all comparisons between e-cigarette emissions and environmental safety limits are invalid.

Additionally, Svarch-Pérez et al. compared PELs expressed in μg per L of working-environment air with e-cigarette aerosol concentrations expressed as μg per L of aerosolized e-liquid. This is a scientifically unjustified comparison. An amount of 1 L of e-liquid represents approximately 200 to 250 days of consumption, based on vaper surveys, which have reported an average daily consumption of 4 to 5 mL [5,6]. In contrast, the PEL concentrations represent exposure from breathing 1 L of air. This corresponds to a maximum of two breaths considering that the average tidal volume for adult humans under resting conditions is 500 mL. Therefore, Svarch-Pérez et al. compared two breaths in an occupational setting with 200–250 days of e-cigarette consumption. In addition to the already fatal flaw of incorrect conversions from ppm to μg/L, such a comparison makes their study discussion and conclusions invalid.

The comparison between tobacco cigarettes and e-cigarettes is also problematic. The authors used data from a Korean study on BTX concentrations in tobacco cigarette smoke [3]. However, that study reported levels in ppb. Thus, Svarch-Pérez et al. made the same error as for PELs when converting ppb values to μg/L. An additional problem is that the reported concentrations represent the amounts of BTX per L of smoke inhaled by a smoker. Considering the average smoke volume per puff of 55 mL (as per the Health Canada Intense puffing regime), 1 L of smoke represents approximately 18 puffs. Thus, the authors compared 18 puffs of a tobacco cigarette (fewer than two cigarettes smoked) with tens of thousands of e-cigarette puffs taken during 200–250 days of use. Therefore, the comparison between tobacco cigarettes and e-cigarettes is also invalid.

Beyond environmental exposure assessment, BTXs are ubiquitous in indoor environments. Benzene is present in indoor spaces at levels of 0.5 to 2.2 µg/m^3^ [7]. The proposed residential maximum exposure limit for benzene is 0.6 μg/m^3^ for long-term exposure [7]. Toluene levels in indoor spaces have been reported to range from 5.5 to 24.7 μg/m^3^, and the recommended residential maximum exposure limit is 2.3 mg/m^3^ [8]. For xylene, the typical levels measured in indoor air range from 0.4 to 17.3 μg/m^3^, while the recommended exposure limit (reference concentration—RfC) is 150 µg/m^3^, adjusted by a total uncertainty factor of 300 from the NOAEL to accommodate for interspecies and intraspecies variation, addressing exposure to more sensitive population subgroups such as children [9].

To understand the magnitude of the errors in the comparisons made by Svarch-Pérez et al., we made the correct unit conversions for toluene and we compared daily exposure from the 20 samples reported by the authors (using an average of 5 mL liquid consumption) with 8 h of exposure in a working environment with toluene at the PEL (10 ppm, 37 μg/mL) using the EPA-defined inhalation rate of 0.027 m^3^/min for moderate activity [10]. We found that daily exposure from e-cigarette use ranged from 0.58 to 653.09 μg, while an 8-h working shift would expose a worker to 488,332.8 μg of toluene. Therefore, e-cigarette use would result in 748 to 844,866-fold lower toluene exposure compared to working in an environment with toluene levels at the PEL. When compared with residential recommended maximum exposure limits (for 24 h of exposure), the respective difference was 30- to 34,380-fold lower exposure to toluene from e-cigarette use, even when underestimating indoor exposure by assuming the unrealistic condition of only a resting respiratory rate for the whole 24 h period (8.6 m^3^/d breathing volume, instead of the guideline-recommended volume of 20 m^3^/d). The respective difference for xylenes was 1.6 to 324-fold lower from e-cigarette use, while only for benzene the differences were from 2-fold lower to 10-fold higher exposure from e-cigarette use compared to the strict residen-tial exposure limits. Such differences are so large that exposure would be far lower from e-cigarette use than from indoor exposure even for population subgroups with lower ventilation rates, such as minors, or with extreme e-cigarette consumption.

Finally, we have detected several potential problems in the analytical methodology and in the results presentation, which we will present only briefly:

1. Tables 2–4 in the original manuscript report BTX concentrations in g/L, which would imply levels hundreds of times above the solubility of these compounds in water. This may have been a typographical error, but such a glaring issue casts serious doubt on the quality control during peer review.
2. The authors collected aerosol from 2 e-cigarette puffs only, a puffing procedure that prevents statistically reliable outcomes (due to inter-puff variability in emissions) and grossly deviates from the recommended experimental standards [11].
3. There is significant ambiguity in the experimental methodology. The use of different columns and extraction techniques (water vs. methanol), the inconsistent descriptions of purge and trap versus thermal desorption, and the lack of procedural detail undermine the reproducibility of the work.
4. The internal standard used for quantification is not identified in the main manuscript. No calibration data is presented.
5. The authors identify a dozen compounds with their retention times (in page 5 of their manuscript), but they had to report two retention times for each compound since they reported having used two columns.

Even if we assume that further and sufficient clarification would be provided by the authors concerning their analytical methodology, the mistakes in converting units from ppm to μg/L, the scientifically unjustified comparison between two breaths in a working environment and 200 to 250 days of e-cigarette consumption, and the erroneous comparisons between tobacco cigarettes and e-cigarettes make a large part of the manuscript, including the results, discussion/interpretation, and conclusions of the study, invalid. Characteristically, five out of the eight paragraphs in the Discussion section and two out of the four paragraphs in the Conclusions section refer to comparisons between BTX exposure from e-cigarettes and tobacco cigarettes or PELs, comparisons which are demonstrably wrong. Based on the above, we believe that the manuscript cannot be corrected and revised. The whole manuscript would need to be rewritten and resubmitted for peer review, focusing only on presenting the analytical results, clarifying the methodological issues we raised, clearly stating that they report amounts per L of aerosolized liquid, and not making comparisons with tobacco cigarettes and PELs. Therefore, we ask the editors to consider retracting the study.
